# The intrinsically disorderly story of Ki-67

**DOI:** 10.1098/rsob.210120

**Published:** 2021-08-11

**Authors:** Lucy Remnant, Natalia Y. Kochanova, Caitlin Reid, Fernanda Cisneros-Soberanis, William C. Earnshaw

**Affiliations:** Wellcome Centre for Cell Biology, University of Edinburgh, ICB, Michael Swann Building, King's Buildings, Max Born Crescent, Edinburgh EH9 3BF, UK

**Keywords:** Ki-67, nucleolus, mitotic chromosome periphery, cell division, mitosis, phase separation

## Abstract

Ki-67 is one of the most famous marker proteins used by histologists to identify proliferating cells. Indeed, over 30 000 articles referring to Ki-67 are listed on PubMed. Here, we review some of the current literature regarding the protein. Despite its clinical importance, our knowledge of the molecular biology and biochemistry of Ki-67 is far from complete, and its exact molecular function(s) remain enigmatic. Furthermore, reports describing Ki-67 function are often contradictory, and it has only recently become clear that this proliferation marker is itself dispensable for cell proliferation. We discuss the unusual organization of the protein and its mRNA and how they relate to various models for its function. In particular, we focus on ways in which the intrinsically disordered structure of Ki-67 might aid in the assembly of the still-mysterious mitotic chromosome periphery compartment by controlling liquid–liquid phase separation of nucleolar proteins and RNAs.

## Graphical abstract

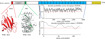

## 1. Introduction to Ki-67

Ki-67 was originally identified as an antigen recognized by a monoclonal antibody created by immunizing mice with nuclei isolated from Hodgkin lymphoma cell line L428 [1]. Cloning and sequencing of the Ki-67 cDNA [2,3] revealed that the amino acid sequence had little similarity to other known proteins. The protein was therefore named after the antibody that identified it. The Ki derives from Kiel (Germany), where the antibodies were developed, with 67 being the well number from the 96-well plate. The entire Ki-67 gene locus was sequenced in 1996 and found to contain approximately 30 000 bases [4].

The original Ki-67 monoclonal antibody was found to stain a specific nuclear sub-structure (subsequently shown to be the nucleolus). This staining was observed in nuclei of cells during G_1_, S and G_2_ phases of the cell cycle but not in cells in G_0_ phase [5]. Given this staining pattern, it was proposed that Ki-67 antibody and protein might be good candidates for evaluating the proliferative status of normal and abnormal human cell populations. Indeed, even in the first study, all proliferating cells tested were positive for Ki-67 staining [1]. Since this initial discovery, several further antibodies, including MIB-1 [6], have been developed against the Ki-67 protein for use as diagnostic tools in the clinical setting. This was necessary as the original antibody failed to stain cells in formalin-fixed paraffin sections, which are normally used in clinical pathology laboratories [7].

Ki-67 is now commonly used as a prognostic marker in the clinic due to its specificity for proliferating cells and ready detection in all cell cycle stages. Positive staining for Ki-67 protein [8–12], alongside other markers [13,14] in a patient tumour sample can be used in grading the primary tumour [15] and metastases [16]. Ki-67 staining has been reported to be of prognostic value in predicting cancer survival rates [9,17,18] and the likelihood of relapse [19]. Many cancer types have been investigated in this way, including non-Hodgkin lymphomas [20], multiple myeloma [21], soft tissue sarcoma [22], prostate cancer [23] and breast cancer [24]. The majority of the greater than 30 000 results on PubMed relate to Ki-67 in this context, while comparatively little work has focused on the cell biology or biochemistry of the protein. Despite the availability of clones and antibodies, the latter studies have yielded a remarkable level of disagreement and controversy.

## 2. Regulation of Ki-67 protein levels

The average level of Ki-67 mRNA and protein in proliferating cells appears to be independent of cell type; similar levels of RNA and protein are seen across several human cell lines [25,26]. Ki-67 levels increase as cells progress through the cell cycle and are controlled by stage-specific regulation of mRNA transcription and protein degradation [25]. Ki-67 protein levels are maximal in mitosis and minimal in late G_1_. The protein half-life is around 90 min [27] so inhibition of protein synthesis for 60 min results in a significant reduction in Ki-67 protein levels [28].

The *MKI67* promotor region contains binding sites for the transcription factor Sp1. Sp1 regulates expression of genes that promote cell cycle progression, including E2F [29], which stimulates Ki-67 transcription [30]. Upon passage of the G_1_ restriction point, CDK4/CDK6 activation triggers phosphorylation of RB which releases E2F [31,32], resulting in increased Ki-67 mRNA transcription. These effects are counter-balanced by continuous degradation of Ki-67 protein in late mitosis and early G_1_ (and G_0_) via the ubiquitin-proteosome system [25,26]. CDK4/CDK6 activation also promotes accumulation of the protein via inhibition of APC/C^Cdh1^ [25]. Thus, in the absence of CDK4/CDK6 activation, cellular pools of Ki-67 protein drop to the extremely low levels characteristic of non-proliferating cells.

## 3. Structural elements of human Ki-67 protein

Ki-67 is encoded in humans by the gene *MKI67.* The Ki-67 cDNA was first cloned by expression screening using the original monoclonal antibody [2]. Analysis of the corresponding transcripts revealed two splice variants [4] encoding two isoforms, both with very large molecular weights (359 and 320 kDa, respectively) and containing a large repetitive region consisting of 16 approximately 360 bp (120 aa) ‘Ki-67 repeats’. Subsequent completion of the cloning and publication of the primary sequence [3] revealed that the shorter variant is missing exon 7. Three additional human splice variants were later identified, all of which have been detected in tissues as well as primary and cultured cells. These variants show characteristic patterns of expression in particular cell types [33]. The significance of these splice variants is currently unknown, however, overexpression of exon 7 (from the longer isoform) in HeLa cells resulted in a reduction in the proliferation rate. The same study reported that overexpression of a fragment of the Ki-67 N-terminus increased cellular proliferation [33].

**Figure 1.**
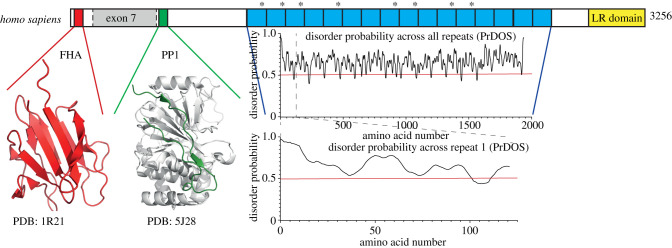
Schematic diagram of human Ki-67. This schematic of Ki-67 isoform I highlights conserved regions. The Ki-67 forkhead associated (FHA) domain (red) is accompanied by its solution NMR structure (PDB:1R21) [37]. Exon 7, highlighted in grey, is missing from isoform II. The PP1-binding domain (green) is accompanied by the crystal structure of the Ki-67 (green):PP1*γ* (grey) holoenzyme complex (PDB:5J28) [35]. The repeat region is highlighted in blue with each individual repeat marked. The FKELF motif, which binds the original Ki-67 monoclonal antibody, is indicated by an asterisk. The disorder probability graphs show disorder across all (top) and the first (bottom) repeat calculated by the PrDOS software [38]. The red line indicates a disorder probability of 0.5. Anything above this is highly likely to be disordered. The LR domain (yellow) is responsible for DNA binding and chromosome association of Ki-67.

Much of the Ki-67 protein is predicted to be unstructured and shows very little cross-species conservation outside of a few conserved functional regions. These recognized structural features include a forkhead-associated (FHA) domain [34], a PP1-binding domain [35], a large region of tandem repeats containing the so-called ‘Ki-67 motif' region and a C-terminal LR (leucine/arginine-rich) chromatin-binding domain [3,36] (figure 1).

The FHA domain is an 11-stranded *β*-sandwich fold that is commonly found in proteins involved in cell cycle regulation [39]. FHA domains recognize phospho-threonine epitopes on proteins [40–42]. The structure of this domain was determined using solution NMR on a bacterially expressed fragment of Ki-67 in 2004 [37] (figure 1).

The PP1-binding motif (RVxF) is conserved in all orthologues of Ki-67, but is not found in the shortest human isoform. This PP1 interacting domain is very similar to that of Repo-Man [43], which is probably derived from a duplication of the Ki-67 gene [44]. *In vivo*, both proteins can bind the *β* and *γ* isoforms of PP1, but not the *α* isoform [35,44]. Repo-Man and Ki-67 target PP1*γ* to anaphase chromosomes through their PP1-binding domains [35,45]. This is required during mitotic exit to reverse mitotic histone phosphorylation [46]. Repo-Man and PP1 are also reported to regulate heterochromatin during interphase; therefore, this domain of Ki-67 could also have roles in heterochromatin maintenance [47,48].

**Figure 2.**
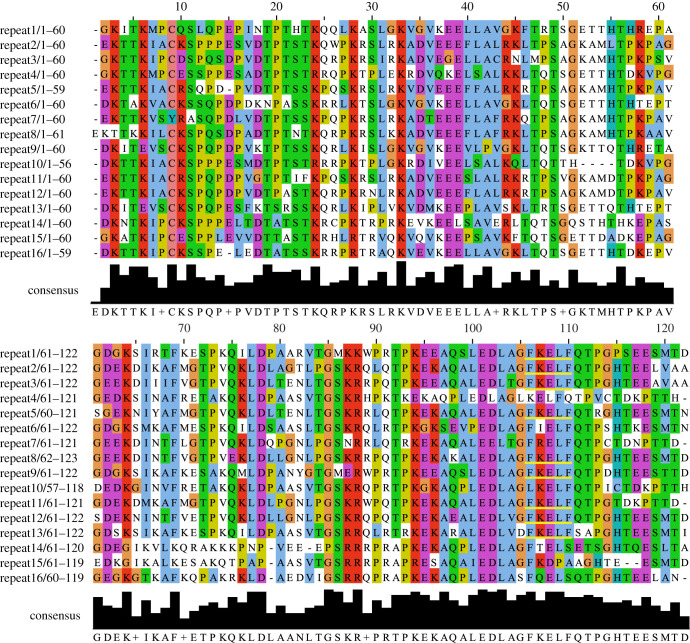
Alignment of the amino acid sequences of human Ki-67 repeats. Sequence alignment of human Ki-67 amino acid repeats in Jalview 2 [49] aligned using ClustalO algorithm [50] and coloured by ClustalX [51]. The consensus sequence is shown at the bottom with a histogram showing the local variation in sequence conservation. The FKELF motif, which binds the original Ki-67 monoclonal antibody is underlined in yellow.

The most unusual structural feature of the Ki-67 protein is the Ki-67 repeat region. This is a region, all encoded within the single huge exon 13, that in human encodes 16 repeats of approximately 120 amino acids. An alignment of the amino acid sequence of the human repeats is shown in figure 2. Within these repeats, there is a highly conserved 22 amino acid sequence (TPKEKAQALEDLAG**FKELF**QTP) known as the Ki-67 motif. This motif contains the epitope to which the original Ki-67 antibody developed by Gerdes binds (**FKELF**) [1]. Remarkably, this allows a single monoclonal antibody to bind nine sites on the protein. The Ki-67 repeat region also contains residues phosphorylated by CDK1 during mitosis [3,52,53]. This repeat region is present in all observed isoforms of human Ki-67 and, as it is contained within a single exon, it is always there in full [33]. A Ki-67 repeat motif is also present in a protein independently identified from rat-kangaroo PtK2 cells named Chmadrin (named because its staining of mitotic chromosomes resembles the striped pattern of Japanese Noh masks). Chmadrin has several regions of structural similarity to Ki-67 as well as a similar subcellular localization [54]. Other vertebrates also contain Ki-67 but, the sequence and number of copies within the repeat region are extremely variable. Interestingly, where present, this region is always found in a single exon.

The first investigation into the evolutionary conservation of Ki-67 was carried out in 1989 [55]. In this study, the original Ki-67 monoclonal antibody was used to determine if the antigen could be detected in other mammalian species. Both normal and transformed proliferating cells were tested and several species (e.g. lamb, calf, dog, rabbit, rat) exhibited positive staining. Ki-67 staining was weakly observed in mouse samples and was absent from others (swine, cat, chicken, pigeon) [55]. At this time, the antigen had still not been cloned, and therefore little work was done to characterize proteins homologous with Ki-67.

**Figure 3.**
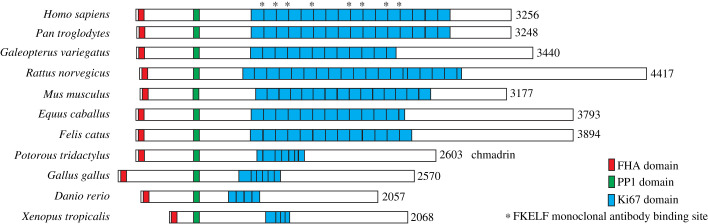
Schematic comparison of Ki67 proteins from several animal species. Comparison of Ki67 proteins from several arbitrarily selected animal species aligned to the PP1-binding domain (PP1 domain). The forkhead associated (FHA) domain (red), PP1-binding (PP1) motif (green) and repeat region (blue) are highlighted.

With modern developments in genome sequencing, it is possible to search for the presence of a Ki-67 motif in many species. The size and number of Ki-67 repeats vary greatly over evolution, and even though nothing was visualized using the original antibody in several species, it is now possible to locate homologous proteins. Ki-67 protein can be identified in all types of vertebrates. Mammalian Ki-67 tends to have a large number of repeats, all of a similar size, however, a Ki-67 gene can also be identified in other tetrapods, including birds, reptiles and amphibians albeit with a lower sequence similarity. Zebrafish (*Danio rerio*) have only four repeats and chickens (*Gallus gallus*) have 9, all of which are much shorter than the repeats in human Ki-67. The organization of Ki-67 protein from a range of arbitrarily selected species is shown in figure 3, with the proteins aligned to the PP1-binding motif. All homologues contain a version of the Ki-67 motif region, but the configuration of this region of the protein is remarkably variable between species. For example, although the chicken has a Ki-67 motif, its sequence is diverged from that in human and the epitope recognized by the original monoclonal antibody is no longer present. This combination of conservation and variability is one of the unanswered enigmas posed by Ki-67.

**Figure 4.**
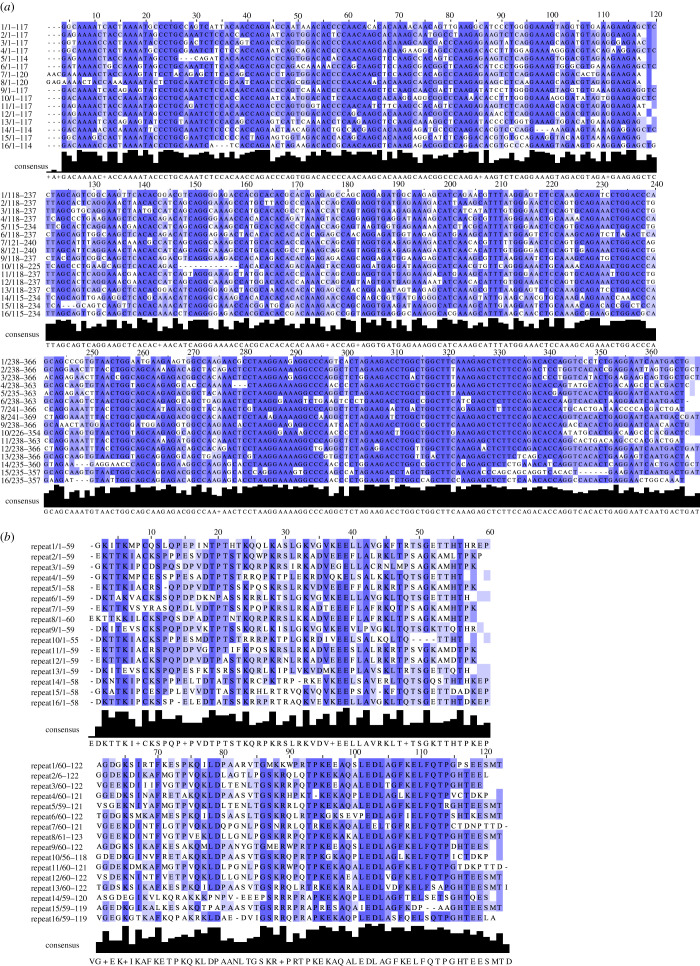
The human Ki-67 repeat DNA sequences are more highly conserved than the corresponding amino acid sequences. (*a*) Alignment of human Ki-67 DNA repeats in Jalview 2 [49] aligned using ClustalO algorithm [50] and coloured by % identity. (*b*) Alignment of human Ki-67 amino acid repeats in Jalview 2 [49] aligned using ClustalO algorithm [50] and coloured by % identity. In both panels, the histogram at the bottom shows the local variation in sequence conservation.

The human Ki-67 repeats are better conserved in their DNA sequence (73 ± 10%) than their amino acid sequence (57 ± 16%) (figure 4). This trend is also observed in other animals, although some vertebrates have relatively higher conservation of their amino acid sequence between repeats. For example, *Gallus gallus* (with 76 ± 15% conservation for the DNA sequence) shows a 65 ± 23% identity for the amino acid sequence of the various repeats. The repeats are also much shorter in chicken, at approximately 60 amino acids, around half the size found in humans. This higher conservation of the DNA sequence than the polypeptide sequence could reflect reduced purifying selection on DNA variants in this region of the protein. Nevertheless, purifying selection has not been absent. Ki-67 repeats are associated with a median *K_a_*/*K_s_* ratio of 0.675 (*n* = 34 comparisons using the software of [56]), less than a ratio of 1.0 which would be indicative of neutral evolution, but also far higher than values for most human protein-coding sequences [57,58]. The *K_a_*/*K_s_* ratio compares amino acid substitutions likely to have little effect on protein function (*K_a_*) to those that are likely to affect function strongly (*K_s_*). This can yield insights into the selective pressure on conserving the protein sequence. We conclude that the repeats have been subject to mild purifying selection, reflecting a moderate tendency to preserve the protein sequence over evolutionary time.

This lower degree of purifying selection could be consistent with the repeat region of Ki-67 being involved in somehow controlling phase separation on the surface of nucleoli and mitotic chromosomes. Intrinsically disordered proteins that are prone to liquid–liquid phase separation generally evolve faster than their non-disordered counterparts [59]. However, the process must be constrained so any amino acid changes are still consistent with promoting phase separation. Thus, this unusual organization of the Ki-67 repeat region could potentially reflect its involvement in forming phase separated condensates.

**Figure 5.**
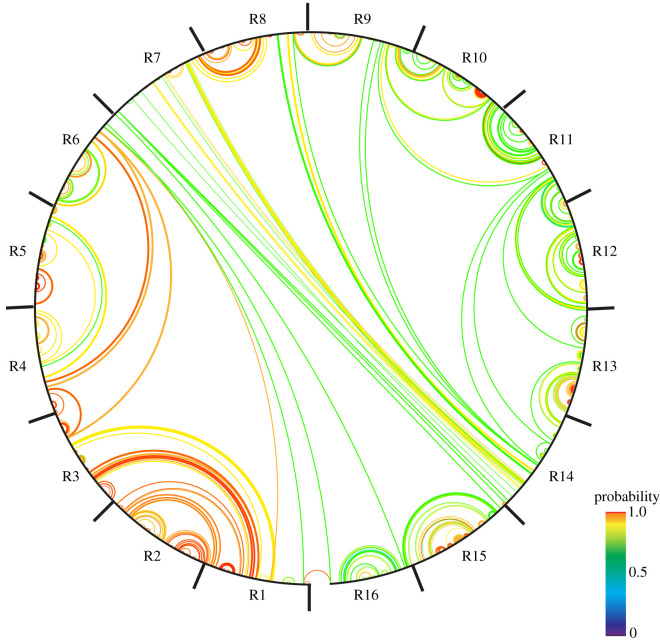
Predicted secondary structure of the human Ki-67 mRNA repeat region. Probability of secondary structure formation (e.g. complementary base-pairing) across the 16 repeats (R1-R16) of human Ki-67 as predicted using the software UNAFOLD [60]. Colours show *p*-values as follows: green: 0.65–0.90; yellow: 0.90–0.99; orange: 0.99–0.999; red: >0.999.

Analysis of the DNA sequence of the repeat region using the software UNAFOLD [60] reveals a high probability for the mRNA to fold into a relatively stable complex secondary structure (figure 5). Although predictions of RNA secondary structures are notoriously unreliable, the predicted ΔG of melting for these folds is similar to that calculated for the 5′ external transcribed spacer of ribosomal RNAs, which are known to have complex secondary structures [61]. The high conservation of the DNA sequences of these repeats suggests that the repeat structure is likely to be maintained by replication slippage facilitated by the sequences being contained in a single exon. Given that different species have differing sequences, but a similar repeat organization, it is possible that there could be some selection to conserve this organization of the mRNA. One highly speculative possibility is that the Ki-67 mRNA may have a conserved function in addition to simply encoding the polypeptide.

The C-terminus of Ki-67 protein including the leucine/arginine-rich (LR domain) [54,62,63] has been shown *in vivo* to be required for Ki-67 to associate with mitotic chromosomes [64,65]. Ki-67 binds to mitotic chromosomes creating a brush-like layer on their surface [65], with the C-terminus associated with the chromatin and the N-terminus extending 66 ± 27 nm out from the surface of the chromosome [66], as discussed below.

## 4. Ki-67 interactions

Mass spectrometry of Ki-67 immunoprecipitates revealed over 400 proteins, including numerous chromatin proteins, many of which are involved in transcription and splicing, as well as proteins involved in pre-rRNA processing, ribosomal biogenesis and protein translation [48]. CDK1, the critical regulatory kinase necessary for mitotic entry, was also present and indeed, Ki-67 has many consensus sites for CDK1 phosphorylation. Ki-67 also interacts with PP1 via its conserved RVxF binding motif [44,48]. PP1 is the principal phosphatase responsible for the reversal of CDK1 phosphorylation during mitotic exit [67–70]. LacI-Ki-67, but not a PP1-non-binding RAXA mutant, can recruit PP1 to an ectopic LacO array. Moreover, PP1*γ* is reduced on anaphase chromosomes upon Ki-67 depletion [44].

Ki-67 interacts with HP1*α*, *β* and *γ* [36,63]. The interaction with HP1*α*, *β* and *γ* has been observed *in vitro* and that with HP1*α* and *β* also in a yeast two-hybrid assay. HP1 overexpression caused Ki-67 to redistribute from nucleoli to heterochromatic foci elsewhere in the nucleus [63]. The interaction between Ki-67 and HP1 occurs via the LR domain of Ki-67 and the chromoshadow domain (CSD) of HP1 [36,63]. These interactions with HP1 may explain the tendency of Ki-67 to associate with constitutive heterochromatin.

Other studies have reported interactions between Ki-67 and Hklp2 (human kinesin-like protein 2) and NIFK (nucleolar protein interacting with the FHA domain of pKi-67), both of which were reported to interact with Ki-67 FHA domain [34,71]. As with HP1, these interactions were identified *in vitro* and in yeast two-hybrid assays. The interaction of NIFK with Ki-67 was reported to occur preferentially in mitotic (rather than interphase) cell-free extracts [34] and NIFK fails to localize to the mitotic chromosome periphery upon Ki-67 depletion [44].

Ki-67, together with several other nucleolar proteins, was reported to be present in immunoprecipitates of CAF-1 p150 from HeLa S3 cells [72]. CAF-1 is the histone chaperone responsible for the co-replicative deposition of histone H3.1 and H3.2 in chromatin [73–76]. This study found that CAF-1 was concentrated in nucleoli based on indirect immunofluorescence [72]. These authors also reported that CAF-1 participates in the recruitment of Ki-67 both to nucleoli and the mitotic chromosome periphery [72,77].

## 5. The localization of Ki-67 across the cell cycle

Ki-67 is present throughout the cell cycle but is not detected in G_0_/quiescent cells. During early G_1_, Ki-67 partially localizes to constitutive heterochromatin associated with satellite DNAs [78]. It then associates with reforming nucleoli with a pattern distinct from either fibrillarin or RNA polymerase I. By mid G_1_, it is concentrated in the newly reformed nucleoli [79], where it remains until the end of G_2_. In HDF and MCF7 cells, Ki-67 was reported to be in the dense fibrillar component [79,80]. However, in most studies with human cancer cell lines, it has been localized to a nucleolar cortex or rim [48,81,82] A recent high throughput immuno-staining study of the nucleolus described 157 other proteins that co-localized with Ki-67 at the nucleolar rim [82].

**Figure 6.**
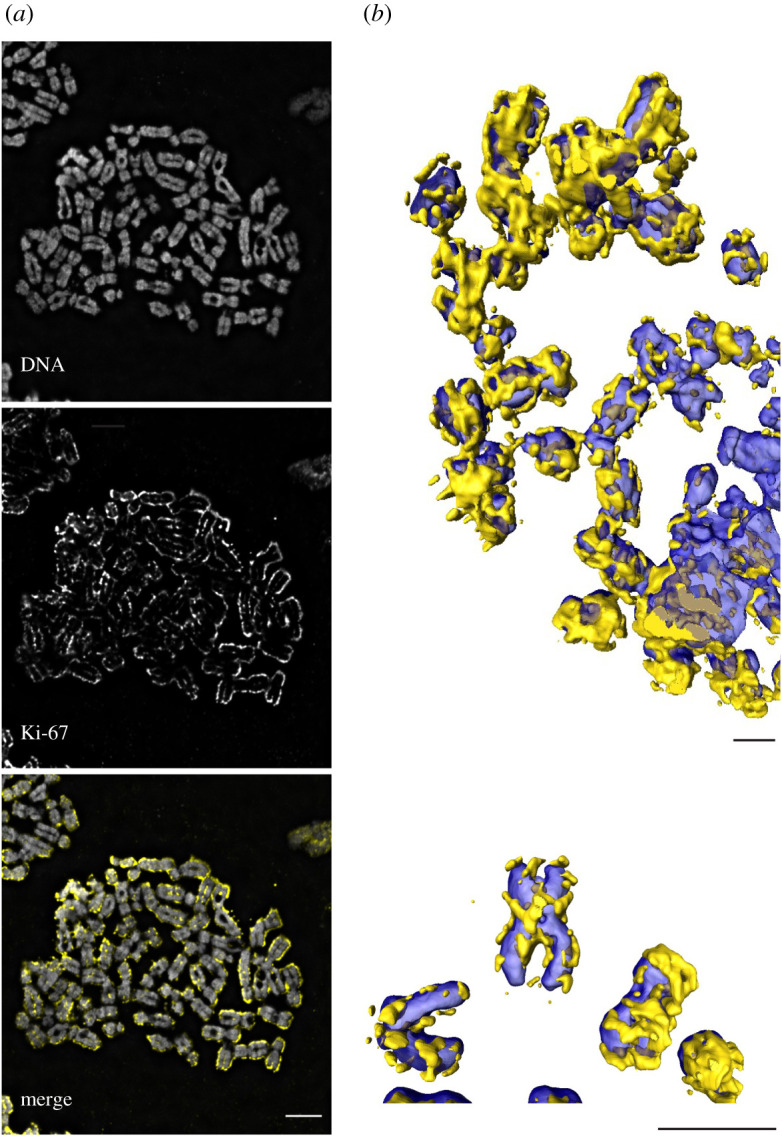
Ki67 is enriched at the chromosome periphery during mitosis. (*a*) Immunofluorescence microscopy of chromosome spread from HeLa CDK1as cells, showing Ki-67 (yellow) enriched on the surface of the DNA (grey). (*b*) Selected chromosomes from a chromosome spread similar to that in A were rendered in three-dimensions using AMIRA, showing how Ki-67 coats the chromosome surface. The discontinuous appearance of the layer is likely to be an artefact of the rendering process in AMIRA. HeLa CDK1as [85] were maintained in DMEM (Invitrogen) supplemented with 5% fetal bovine serum (Invitrogen) and 100 U/ml penicillin G and 100 µg ml^−1^ streptomycin sulphate (Invitrogen). Colcemid was added to the culture at a final concentration of 0.1 µg ml^−1^ for 75 min before harvesting by mitotic shake off. Cells were treated with hypotonic solution (75 mM KCl) for 10 min before Cytospin at 1900 RPM, High intensity for 10 min. Following fixation with 4% paraformaldehyde for 10 min at 37°C, immunofluorescence of metaphase chromosomes was carried out using Anti-Ki-67 mouse monoclonal antibody 1 : 500 (9449S, Cell Signalling) and Alexa 555 1 : 1000. Chromosomes were mounted with VECTASHIELD Antifade Mounting Medium with DAPI. Scale bar, 5 mm.

The greatest change in Ki-67 localization occurs during prophase when it moves away from the nucleolus. By late prophase, it is detectable on the surface of newly forming chromosomes, where it remains throughout mitosis until telophase. There it is localized to a compartment referred to as the mitotic chromosome periphery compartment (MCPC), or perichromosomal layer [83,84] (figure 6). The MCPC consists of proteins and RNAs that comprise more than a third of mitotic chromosome volume and up to a third of mitotic chromosome mass [86]. In one study of human and mouse mitotic chromosome spreads, Ki-67 immunostaining was excluded from centromeric and nucleolar organizer regions, as well as pericentromeric heterochromatin as defined by the mouse major satellite [87]. Relocalization of Ki-67 protein away from the mitotic chromosomal surface and into pre-nucleolar bodies was reported to start during telophase as the G_1_ nuclei start to reassemble [84].

Many proteins belonging to the MCPC are nucleolar in interphase [86]. A recent study described 65 nucleolar proteins that localize to the mitotic chromosomal periphery at various times in mitosis. Two major recruitment patterns were observed: early (during prometaphase, 46 proteins) and late (after metaphase, 19 proteins). Interestingly, 49 of these 65 proteins localized to the nucleolar rim in interphase together with Ki-67 [82]. In this study, live-cell imaging also detected a sub-population of exogenous GFP-tagged Ki-67 protein near the nuclear periphery.

The changes in Ki-67 localization when cells transition from G_2_ to mitosis are likely to result from hyperphosphorylation, which can be detected by a change in the electrophoretic mobility of Ki-67 [52,64]. The Ki-67 sequence contains 90 CDK1 traditional consensus target sequences, of which 70 are in the repeat region [3,52,53]. Indeed, Ki-67 immunoprecipitates were reported to contain CDK1 [48]. Immunoblotting studies also reveal that mitosis-specific phosphorylated bands of Ki-67 react with the MPM-2 antibody [52,88,89]. This could be due to the action of any of the several reported MPM-2 kinases, including casein kinase 2 [90], MAP kinase [91], Cdk1 (Cdc2), Polo/Plk [92,93] and NIMA [94].

Ki-67 remains phosphorylated throughout mitosis and is then dephosphorylated by PP1 during anaphase/telophase [45]. This may help to promote the dissociation of the chromosome periphery [45,62]. It may also trigger the aggregation of chromosomes, which has recently been shown to be involved in excluding large particles such as ribosomes from the inter-chromosomal space and therefore, from the daughter nuclei [66].

## 6. Controversies concerning the effects of Ki-67 depletion in cells and organisms

There have been several investigations into the effect of depleting Ki-67 protein from cells and organisms using a range of techniques including antisense oligonucleotides, RNAi and classical/CRISPR gene targeting. These studies report a diverse range of often contradictory results. Ki-67 depletion has been variously reported to result in cell death, reduced cell proliferation or have no significant defect, depending on the method used and cell type studied.

### 6.1. Reduced proliferation/cell death

There appears to be a consensus that Ki-67 antisense oligonucleotide (ASO) treatment results in inhibition of cell proliferation and/or increased cell death. This was tested with similar results in various cell lines, including IM-9 [3], RT-4, MCF-7, RM-11 and MB-49 [95]. IM-9 and RT-4 also exhibited increased levels of apoptosis following Ki-67 ASO treatment [95]. Exposure of 786-0 (human renal carcinoma) cells to siRNAs targeted against Ki-67 resulted in an inhibition of cell growth and increased apoptosis [96]. Similar phenotypes were also observed after shRNA treatment in these cells [97]. One possible weakness of these studies is that none have showed a rescue of the phenotype using ASO/siRNA/shRNA-resistant Ki-67 cDNAs.

In an orthogonal approach, microinjection of anti-Ki-67 antibodies into mouse Swiss 3T3 cells also inhibited cell proliferation, apparently by blocking the cell cycle progression in the following mitosis [98].

As a follow-up to those earlier findings and attempt to resolve controversies (see below), a more recent study attempted to examine Ki-67 function in several cell types. Depletion of Ki-67 by siRNA resulted in a reduction in the number of cells in S-phase for hTERT-RPE-1, WI-38, IMR90 and hTERT-BJ [99]. This effect was only seen in cells that induced expression of p21 in response to the reduction in Ki-67. The pathway linking Ki-67 depletion to p21 expression/accumulation remains to be identified, although it is tempting to speculate that Ki-67 depletion may activate p53 via a nucleolar stress response [100,101].

### 6.2. No obvious phenotypic consequences

In another recent study, shRNA-mediated knockdown of Ki-67 in U2OS, HeLa and BJ-hTERT cells had no obvious effect on the proliferation of these cells [48]. Furthermore, shRNA depletion of Ki-67 in NIH-3T3 cells did not affect their ability to re-enter the cell cycle after serum starvation. Finally, and most convincingly, a Ki-67 knockout mouse was created using TALEN-mediated gene targeting. The authors looked carefully for residual Ki-67 protein and failed to detect any. These Ki-67 mutant mice developed normally and MEFs from the mice proliferated normally [48].

Further support for the non-essential nature of Ki-67 was obtained when Ki-67 was depleted in MCF-10A and DLD-1 cells by insertion of a premature nonsense (STOP) mutation into the first coding exon [102]. This Ki-67 gene disruption was not lethal and did not result in chromosomal instability. Likewise, HeLa cells in which the Ki-67 gene was disrupted by CRISPR/Cas9 targeting continued to proliferate, although some differences in mitotic progression could be observed in the live-cell analysis [65,103]. By contrast, mitotic spreads of chromosomes from HCT116 cells did not display any obvious abnormalities following Ki-67 depletion [104]. However, 3D-CLEM analysis of Ki-67 depleted chromosomes did show an apparent reduction in chromosomal volume in hTERT-RPE-1 cells [86].

Perhaps the definitive evidence for a lack of an essential role of Ki-67 in cancer cells comes from a recent analysis of data using the Dependency Mapper of the Cancer Dependency Map Project. There, the loss of Ki-67 protein was found to have no effect on the proliferation in 725 out of 739 cell lines [105]. The loss of Ki-67 was found to correlate with broad changes in the levels of thousands of transcripts in several different cell lines [105]. This was interpreted as evidence for Ki-67 in establishing general chromatin states, rather than playing specific roles in the regulation of particular genes. This hypothesis was consistent with previous results from the same group, indicating that Ki-67 can interact with a wide range of factors involved in modifying chromatin states [48]. In the more recent study, the authors went on to show that although Ki-67 protein is not required for cell proliferation, in its absence prominently affects the behaviour of cancer cell lines introduced into immune-compromised mouse models [105]. There, lack of Ki-67 correlated with impaired tumorigenesis and an impaired ability of injected tumour cells to metastasize.

Despite several decades of controversy, it now appears clear with the advent of CRISPR/Cas9 technology that the Ki-67 protein is not essential for life. Why antisense oligonucleotides can reportedly kill cells remains to be determined. However, the huge size of the Ki-67 mRNA has made it difficult to construct rescue cDNAs to confirm the specificity of oligonucleotide-based targeting constructs.

## 7. Roles of Ki-67 in the nucleolus and surrounding heterochromatin

The nucleolus was the first nuclear subdomain to be recognized [106]. Nucleoli are multifunctional compartments in which ribosomal RNAs are transcribed, processed and assembled into mature ribosomes [107–110]. Other RNPs, including signal recognition particle (SRP) and telomerase are also matured within nucleoli [111]. These functional events are associated with a characteristic substructure of the nucleolus, which has recently been described as a multi-phase liquid condensate [112,113]. rRNA transcription occurs in and around the *fibrillar centres*. These transcripts are then initially processed in the *dense fibrillar component* and finally matured and assembled into ribosomes in the *granular component* [114,115]. More recently, a number of components of a fourth nucleolar compartment—the *nucleolar cortex*—has been described [82].

Although it is clear that Ki-67 localizes to nucleoli, there remains some controversy over whether it is required for rRNA production. In support of such a link, Ki-67 was found to physically associate with the promoter and gene body of the rDNA cluster [116]. Ki-67 also co-immunoprecipitates with proteins involved in ribosome biogenesis [48]. Furthermore, Ki-67 depletion leads to a loss of association of chromosome 17 alpha-satellite sequences [72] and chromosome 13p [44] with nucleoli. This is accompanied by increased transcription of alpha-satellite DNA, suggesting that centromeres move away from the repressive perinucleolar environment [103]. The latter is consistent with nucleolar inactivation (or a failure of nucleolar re-activation after mitotic exit) upon Ki-67 depletion since both loci are physically proximal to the rDNA on those respective chromosomes. Finally, nucleolar morphology was found to be abnormal upon Ki-67 RNAi and levels of 47S pre-ribosomal RNA transcripts were decreased [44]. However, a later study reported Ki-67 to be dispensable for pre-rRNA production [48]. The reason for these differing conclusions is not clear, and the connections between Ki-67 and rRNA production and processing merit further exploration.

Nucleoli are typically surrounded by constitutive heterochromatin characterized by the histone marks H3K9me2/3 and H4K20me3 [117–120], as well as their readers HP1*α* and Su(var)3-9 proteins. HP1*α* binds via its chromodomain to H3K9me3 [121,122] and interacts with the H3K9 methyltransferase Su(var)3-9 via its chromo shadow domain (CSD) [123,124]. Su(var)3-9 anchored via HP1*α* to H3K9me3 nucleosomes can modify neighbouring nucleosomes, thus creating an epigenetic loop that can locally spread heterochromatin [124]. Constitutive heterochromatin is enriched on α-satellite DNA at pericentromeres and at telomeres, as well as other regions where transcription is generally silent, e.g. near the nuclear periphery [125,126].

Apart from any effect on rRNA production, Ki-67 was reported to functionally interact with constitutive heterochromatin near nucleoli. Ki-67 depletion caused a decrease in peri-nucleolar levels of H3K9me3 and H4K20me3, which were redistributed to foci scattered throughout the nucleoplasm. Paradoxically, cellular levels of perinucleolar HP1 proteins were unchanged and overall levels of H3K9me3 and H4K20me3 also remained constant following Ki-67 depletion [48]. Interestingly, the same study reported that peri-nucleolar chromatin compaction was reduced upon Ki-67 depletion, as measured by FLIM-FRET of H2B-GFP/H2B-mCherry [48]. These effects of Ki-67 on heterochromatin were reported to be dosage-dependant. Overexpression of *Xenopus* Ki-67 in human cancer cells caused ectopic heterochromatin formation, visualized as areas of more intense DNA staining associated with local concentrations of H3K9me3 [48]. Overexpression of the LR-domain, which is required for chromatin binding of Ki-67, resulted in chromatin hyper-condensation as visualized by DNA staining [36,54]. Interestingly, this domain is necessary for Ki-67's interaction with HP1 [36,63], although the mechanism for the hyper-condensation remains to be determined.

In addition to being functionally linked with peri-nucleolar constitutive heterochromatin, Ki-67 was reported to influence facultative heterochromatin on the X-chromosome, but only when that chromosome was located adjacent to the nucleolus [99]. Ki-67 depletion led to an apparent decrease in H4K20me1 and H3K27me3 levels on the peri-nucleolar inactive X, which tended to show an increased tendency to localize to the nuclear periphery [99].

Together, these results suggest that Ki-67, and possibly also other components of the nucleolar cortex have a role in establishing a compartment of transcriptionally inactive chromatin that coats the surface of nucleoli [127]. The role of this peri-nucleolar heterochromatin compartment is not known. The proteins of the nucleolar cortex are enriched in intrinsically disordered domains, a feature that has been associated with phase separation, often in association with RNAs [82,128–130]. Indeed, heterochromatin was one of the first chromatin types to be shown to be able to undergo phase separation *in vitro* [131–134]. It is thus possible that Ki-67 and other cortex components merge with heterochromatin to establish a phase surrounding nucleoli. Such a phase could help to insulate nucleoli from the surrounding euchromatin of the bulk nucleoplasm.

## 8. Roles of Ki-67, topoisomerase IIα and condensin in mitotic chromosome structure

Acute depletion of condensin subunit SMC2 in human HCT116 cells produces a severe chromosome clumping phenotype; however, the chromosome periphery is not perturbed [104,135]. By contrast, simultaneous acute depletion of Ki-67 plus total condensin (SMC2 depletion), but not depletion of condensin I or II alone (depletion of CAP-H or CAP-H2, respectively) in HCT116 cells gives rise to a so-called ‘rice cake' phenotype, where mitotic chromosomes become a contiguous mass, in which separate chromosomes cannot be distinguished [104]. As in the case of the chromosome aggregation phenotype seen after Ki-67 depletion [44,65] this ‘rice cake' structure appears only after nuclear envelope breakdown [104].

The ‘rice cake' is often observed at one side of the cytoplasm, with HOECHST-stained protrusions projecting towards the other side of the cell [104]. These protrusions contain Topoisomerase IIα, CENP-A, CENP-I, NDC80 and BubR1 [104]. Similar protrusions were previously seen in SMC2-depleted cells [135–137]. Loss of condensin does not abolish kinetochore formation or activity, but does reduce the compliance of pericentromeric heterochromatin. Thus, kinetochores can attach to microtubules and undergo ‘excursions’ in which they move away from the body of the chromosomes, which stretch, forming thin fibres. These observations suggest that Ki-67 is not required for assembly of a functional kinetochore [104].

In the more recent study, Ki-67 acute depletion did not perturb SMC2 localization to mitotic chromosomes, based on indirect immunofluorescence [104]. This is consistent with a previous report in HCT116 cells, where SMC2 and CAP-H localization to mitotic chromosomes was not changed upon Ki-67 acute depletion [138]. By contrast, the association of CAP-H2 (always) and Topoisomerase IIα (depending on the staining protocol) with the mitotic chromosome axis was reduced [138]. Furthermore, Ki-67 was found to reciprocally co-immunoprecipitate with Topoisomerase IIα from mitotic cell extracts, leading the authors to suggest that Ki-67 might influence the mode of Topoisomerase IIα association with the mitotic chromosome scaffold [138]. In a previous study, Ki-67 depletion by RNAi in HeLa cells did not influence KIF4 localization on mitotic chromosomes and did not impair mitotic chromosomal architecture, as probed using the intrinsic metaphase structure (IMS) assay upon Ki-67 depletion [44].

In the more recent study, a supervised machine-learning algorithm could separate DNA-stained images of control, Ki-67-depleted, SMC2-depleted and Ki-67/SMC2-depleted cells into 4 distinct classes. This suggests that either depletion of either SMC2 or Ki-67 or both together results in a unique phenotype [104]. Taken together, these results suggest that Ki-67 may cooperate with both condensin I and II to influence the structure of chromosomes in mitosis.

## 9. Ki-67 organizes the mitotic chromosome periphery compartment

Ki-67 binds to the mitotic chromosome periphery via its C-terminal LR domain [65], with the N-terminal end of the protein projecting outwards 66 ± 27 nm into the cytoplasm forming an extended molecular brush-like arrangement [66]. Upon treatment with the CDK inhibitor flavopiridol, which induces mitotic exit accompanied by clustering of the chromosomes, this brush collapses to about 50% of its prometaphase length [66]. Interestingly, GFP-Ki-67 is highly mobile during prometaphase in FRAP experiments, with a recovery time of approximately 12 s and a mobile fraction of 90%. This mobility drops approximately fivefold after the onset of anaphase with the mobile fraction decreasing to approximately 30% [64]. Throughout all of this, Ki-67 remains localized to the MCPC. Interestingly, the Ki-67 mobility drops a further approximately twofold in early G_1_ after nucleolar reformation.

FCS measurements using endogenously tagged Ki-67 suggested that about 270 000 Ki-67 molecules bind to mitotic chromosomes in HeLa cells [65]. The authors calculated that this would yield a density of 210 Ki-67 molecules/µm^2^, although it is not clear how they calculated the surface area of HeLa mitotic chromosomes. Remarkably, this high density of Ki-67 is precisely conserved in chicken DT-40 cells. IBAQ analysis of proteomics data from isolated mitotic chromosomes yields 89 000 Ki-67 molecules per DT40 cell (I. Samejima 2015, unpublished, based on datasets of [139]). With a chromosome surface area of 424 µm^2^ measured by serial block-face scanning electron microscopy [135], this yields 210 Ki-67 molecules/µm^2^ in metaphase DT40 cells.

Ki-67 is apparently required to recruit all known components of the MCPC to the chromosome surface in mitosis [44,82]. This was first demonstrated by staining for a number of components of the MCPC and by quantitating density profiles at the chromosome periphery in electron micrographs of mitotic cells [44,82]. More recently, of 61 MCPC proteins tested in HeLa cells, none was able to fully localize to the mitotic chromosome periphery upon Ki-67 depletion [82]. In the absence of Ki-67, the MCPC components form aggregates in the mitotic cell cytoplasm [44,82]. Although it has yet to be formally demonstrated, these aggregates almost certainly reflect LLPS by the MCPC components. Curiously, when examined by correlative light and electron microscopy, the aggregates were indistinguishable from bulk cytoplasm [44,82].

The function of Ki-67 in the MCPC remains enigmatic. It is clear that Ki-67 is required to keep chromosomes individualized after nuclear envelope breakdown. This led to the proposal that Ki-67 functions as a surfactant to disperse mitotic chromosomes [65]. Upon Ki-67 depletion, chromosomes form as individual bodies during prophase, often on the inner surface of the nuclear envelope. However, they become tightly clustered into one or more clumps following nuclear envelope breakdown [44,65,82,86]. In another study, double acute depletion of condensin and Ki-67 in HCT116 cells gave rise to the so-called ‘rice cake' phenotype, with the mitotic chromosomes forming a single contiguous mass [104]. Paradoxically, despite these striking abnormalities, cells survive Ki-67 depletion by siRNAs and shRNAs and disruption of the Ki-67 gene [48,65,99,103]. Live cell imaging of HeLa Ki-67 knockout cells revealed problems with chromosome congression and with anaphase onset after release from a nocodazole arrest; however, the cells lacking Ki-67 survived and continued to proliferate [65,103]. The underlying survival mechanism remains to be determined.

Chromosome clumping following Ki-67 depletion can be avoided by overexpressing the Ki-67 LR domain, provided that it is fused to any of several other parts of Ki-67 [65]. The effect of chromosome individualization is dosage-dependent since, upon Ki-67 overexpression, chromosomes are spaced further apart [65]. Overexpression of positively charged histones also partially rescues Ki-67 depletion phenotype [65], leading to the notion that the MCPC establishes a net positive charge on the surface of chromosomes, so that electrostatic repulsion may help to keep them separate. However, this would seem to contrast with the observation that the Ki-67 molecular brush collapses during mitotic exit, at which point it seems to actively promote aggregation of the segregating chromosomes into a tight mass. This timing plus the observation that Ki-67 has been described to associate with CDK1 would tend to suggest that phosphorylation of the protein may be required for its surfactant function in early mitosis. Under this scenario, the differences in Ki-67 organization and function after anaphase onset would be likely to depend at least in part on dephosphorylation, possibly by PP1 recruited to its binding site on Ki-67.

In contrast to its role in chromosome dispersion during early mitosis, Ki-67 has been proposed to play a role in the exclusion of cytoplasmic components during nuclear reformation at the end of mitosis [66]. Upon Ki-67 depletion, ribosomes, as well as ectopically expressed genetically encoded multimeric nanoparticles, are interspersed between chromosomes and end up inside the reforming nucleus [66]. By contrast, in the presence of Ki-67, the chromosomes cluster more tightly together during mitotic exit, with the result that these large particles are excluded from the inter-chromosomal space, and therefore, from the newly reformed nucleus [66].

A third possible function for Ki-67 and the MCPC is as a site where key nucleolar components are concentrated so that they end up inside the nuclear envelope as cells exit mitosis. Indeed, the volume of the MCPC roughly equals the nucleolar volume [86]. By concentrating rRNA processing factors with pre-rRNAs and other nucleolar proteins on the chromosome surface, cells would avoid the need to transport these components into the nucleus to re-activate its nucleoli and begin ribosome biogenesis in the next cell cycle. Since nuclear pore formation and maturation occurs with a time lag after nuclear envelope assembly [140,141], this would avoid a kinetic bottleneck for cell growth and homeostasis. Indeed, in one study, nucleolar reactivation after mitosis and rRNA synthesis was found to be impaired following Ki-67 depletion [44]. By contrast, a later study reported Ki-67 to be dispensable for pre-rRNA production [48]. Further investigations are needed to explain this contradiction.

## 10. Is Ki-67 a regulator of liquid–liquid phase separation?

In cells depleted of Ki-67, components that are normally associated with the mitotic chromosome periphery mis-localize and form large aggregates in the cytosol [44]. Strikingly, these aggregates often localize at one end of the metaphase plate [44]. This could result from passive pushing by microtubules extending from the spindle poles. The aggregates are not detected by SBS-SEM in 3D-CLEM experiments, suggesting that their density resembles that of bulk cytoplasm [44].

Although this remains to be experimentally proven, we speculate that these aggregates are likely to be formed by liquid–liquid phase separation (LLPS). Intrinsically disordered regions are a common feature of proteins that undergo LLPS. In a recent study, many proteins of mitotic chromosomes [84,86,142–147] were predicted to be disordered using the protein disorder prediction tool IUPred2A [82,148]. Indeed, at least 81% of the MCPC proteome was predicted to have at least one long disordered domain [82].

Prominent among those proteins with disordered domains is Ki-67. As shown in figure 1, much of the Ki-67 repeat region is predicted to be intrinsically disordered. This suggests a mechanism by which Ki-67 might recruit the proteins and RNAs of the MCPC. Tethering the C-terminus of the protein to the chromosome surface would potentially create a region coating the chromosomes with a high potential to undergo LLPS. This might explain why no single region of Ki-67 (other than the chromatin-binding motif) is required for its function in keeping mitotic chromosomes separate. It may be that rather than acting as a surfactant, as previously proposed, Ki-67 on the chromosome surface might act more like a detergent, ‘dissolving' many nucleolar proteins and RNAs (which also undergo phase separation).

The above predicts that the chromosome periphery, rather than being a structured protein:RNA assembly may be a tethered liquid-like phase, at least in early mitosis. This would be consistent with the high mobility of periphery components observed by FRAP. It would also suggest that components of liquid-like domains such as nucleoli and heterochromatin) [112,113,131–134] may retain their phase separation behaviour even during mitosis. Indeed, a brief treatment of mitotic cells with 1,6-hexanediol (a known disruptor of many liquid-like phases) causes a rapid disruption of the MCPC (F.C.-S. 2021, unpublished).

As described above, FRAP data suggest that the MCPC layer may become more static during mitotic exit. This is consistent with recent experiments suggesting that Ki-67 undergoes a functional switch when CDK1 activity starts to decline causing the molecular brush to collapse. Also at this time, pre-nucleolar bodies begin to form, possibly reflecting the release of nucleolar components from the MCPC.

## 11. Perspectives

Despite being one of the most highly cited of cellular proteins, many aspects of Ki-67 function remain to be explained. What explains the remarkable mixture of conserved overall organization but highly divergent detailed structure of the single-exon repeat region? Does Ki-67 function in organization of the peri-nucleolar heterochromatin and mitotic chromosome periphery compartment (MCPC) by locally regulating and organizing LLPS of chromatin and nucleolar proteins? Is this how it modulates the expression of thousands of genes in cancer cells and is this its main function? And what is the cellular function of the MCPC? Does Ki-67 or the MCPC have a role in promoting nucleolar re-activation and efficient rRNA production after cell division? The availability of specific reagents and development of methods for working with huge proteins and genes should ultimately allow us to assemble a more orderly picture of the role of this fascinating protein.

## Data accessibility

This article has no additional data.
